# *H*_18_ Carbon: A New Metallic Phase with *sp*^2^-*sp*^3^ Hybridized Bonding Network

**DOI:** 10.1038/srep21879

**Published:** 2016-02-23

**Authors:** Chun-Xiang Zhao, Chun-Yao Niu, Zhi-Jie Qin, Xiao Yan Ren, Jian-Tao Wang, Jun-Hyung Cho, Yu Jia

**Affiliations:** 1 International Laboratory for Quantum Functional Materials of Henan, and School of Physics and Engineering, Zhengzhou University, Zhengzhou 450001, China; 2 Center for Advanced Analysis and Computional Science, Zhengzhou University, Zhengzhou 450001, China; 3 Beijing National Laboratory for Condensed Matter Physics, Institute of Physics, Chinese Academy of Sciences, Beijing 100190, China; 4 Department of Physics and Research Institute for Natural Sciences, Hanyang University, 17 Haengdang-Dong, Seongdong-Ku, Seoul 133-791, Korea

## Abstract

Design and synthesis of three-dimensional metallic carbons are currently one of the hot issues in contemporary condensed matter physics because of their fascinating properties. Here, based on first-principles calculations, we discover a novel stable metallic carbon allotrope (termed *H*_18_ carbon) in 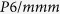 (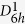) symmetry with a mixed *sp*^2^-*sp*^3^ hybridized bonding network. The dynamical stability of *H*_18_ carbon is verified by phonon mode analysis and molecular dynamics simulations, and its mechanical stability is analyzed by elastic constants, bulk modulus, and shear modulus. By simulating the x-ray diffraction patterns, we propose that *H*_18_ carbon would be one of the unidentified carbon phases observed in recent detonation experiments. The analysis of the band structure and density of states reveal that this new carbon phase has a metallic feature mainly due to the C atoms with *sp*^2^ hybridization. This novel 3D metallic carbon phase is anticipated to be useful for practical applications such as electronic and mechanical devices.

Carbon has a variety of structural allotropes due to its ability of different hybridizations^1^. It is well known that there exist three carbon allotropes in natural materials, that is, graphite, diamond, and amorphous carbon, containing the *sp*^2^, *sp*^3^, and mixed *sp*^2^/*sp*^3^ hybridized carbon atoms^2^, respectively. In the past three decades, intensive theoretical and experimental efforts have been focused on synthesizing new allotropes of carbon, among which, the zero-dimensional (0D) fullerenes^3^, one-dimensional (1D) carbon nanotubes^4^, and two-dimensional (2D) graphene^5^ are the three most prototypical examples. So far, many new carbon allotropes such as 1D *sp*-carbyne, 2D *sp*-*sp*^2^-graphyne, and three-dimensional (3D) *sp*-*sp*^3^-yne-diamond have been experimentally fabricated or theoretically predicted^6^,^7^, most of them exhibit their intriguing mechanical and electronic properties. Recently, a new carbon allotrope with hardness even higher than diamond has also been observed by compressing graphite with pressure over 17 GPa^8^. Motivated by this experimental finding, several carbon crystalline phases such as monoclinic *M*-carbon^9^, bct-C_4_ carbon^10^, *W*-carbon^11^, *O*-carbon^12^, and *Z*-carbon^13^ were proposed to simulate this high-pressure carbon phase. These prototypical examples have given rise to significant impacts in material and information sciences, stimulating experimental and theoretical attentions on carbon allotropes^14^,^15^,^16^,^17^,^18^,^19^,^20^,^21^,^22^,^23^.

Among various types of carbon materials, metallic carbon allotropes exhibit more fascinating properties, e.g., a highly efficient catalytic property due to its high electronic density of states (DOS) at the Fermi level^24^. It was also identified that metallic carbon becomes magnetic when the Stoner-like criterion^25^,^26^ is satisfied. Furthermore, metallic carbon showed a number of intriguing properties such as phonon-plasmon coupling^27^, superconductivity^28^,^29^ and negative differential resistance^30^. Consequently, the exploration of metallic carbon candidates have attracted considerable attention in the synthesis and design of new carbon allotropes. However, all of the 3D carbon allotropes mentioned above are semiconductors or insulators.

There have so far been few progresses in search of 3D metallic carbon. Some hypothetical 3D carbon allotropes such as ThSi_2_-type tetragonal carbon^31^, hexagonal H-6 carbon^32^, and *K*_4_ carbon^33^ have been proposed to be metallic. However, all of such structures were found to be dynamically unstable^19^,^34^,^35^. In 2012, a simple cubic phase of carbon was reported to be metallic under 3 TPa, but it becomes unstable when pressure is removed^36^. Recently, a 3,4-connected T6 carbon allotrope was proposed to be metallic, but it was also unstable at temperature about 500 K^37^. More recently, a 3D metallic *K*_6_ carbon with pure *sp*^3^ hybridization was reported to have a high DOS at the Fermi level^38^, however, its stability is too low to be synthesized. To our knowledge, all of these theoretical predictions of 3D metallic carbon allotropes have not been experimentally realized so far.

Here, based on first-principles total-energy and phonon calculations^39^,^40^,^41^,^42^,^43^,^44^,^45^,^46^, we discover a new stable 3D metallic carbon allotrope in 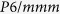 (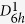) symmetry with a mixed *sp*^2^-*sp*^3^ hybridized bonding network. This new phase is composed of eighteen atoms per hexagonal primitive cell (hereafter termed *H*_18_ carbon), having a larger atom density of 3.135 g/cm^3^ compared to 2.28 g/cm^3^ for graphite. The calculated elastic constants show that the *H*_18_ carbon is a brittle material. From the analysis of the phonon spectra, we find that *H*_18_ carbon does not have any unstable vibration modes. Interestingly, the simulated x-ray diffraction (XRD) pattern of *H*_18_ carbon matches one of the unidentified carbon phases observed in recent detonation experiments^47^. The calculated band structure and DOS of *H*_18_ carbon manifest a metallic feature mainly due to the C atoms with *sp*^2^ hybridization. The *H*_18_ carbon has great potential application in electronics, mechanics, and some other related fields due to its novel properties.

## Results

**Figure 1:**
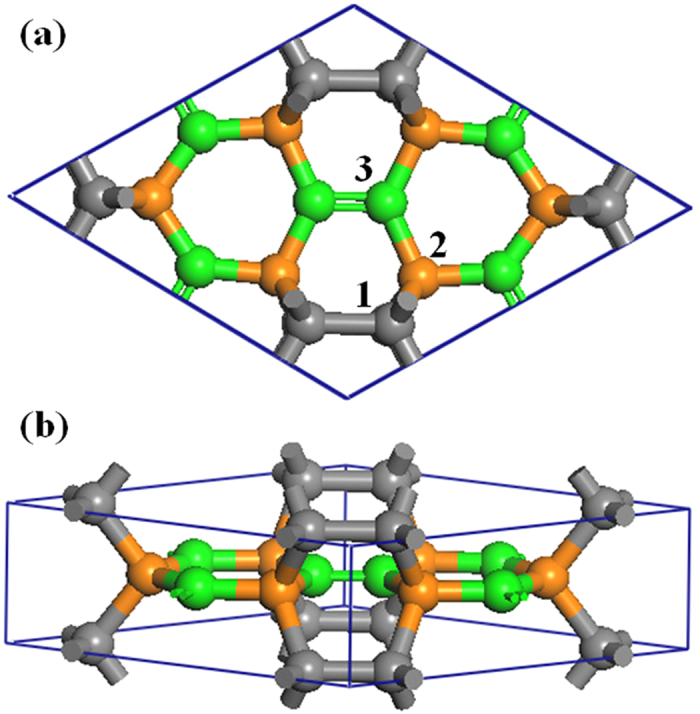
Top (**a**) and side (**b**) views of *H*_18_ carbon in 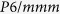 (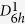) symmetry with single and double bonds. The carbon atoms on 6 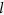 (0.2579, 0.1289, 0), 6 m (0.583, 0.7915, 0.5), and 6 m (0.8933, 0.4467, 0.5) Wyckoff positions are denoted by C_1_ (gray), C_2_ (orange) and C_3_ (green), respectively.

Figure. 1 shows the structure of *H*_18_ carbon with a 3D 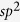-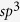 hybridized bonding network in 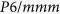
(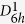) symmetry. Here, the hexagonal primitive cell contains eighteen C atoms with equilibrium lattice parameters a = b = 7.125 Å, c = 2.605 Å. The C_1_, C_2_, and C_3_ atoms in Fig. 1 are bonded to four, four, and three neighboring carbon atoms, thus forming 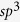, 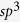 and 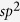 bonds, respectively. The calculated bond lengths 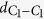, 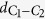, 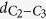, and 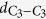 are 1.591, 1.631, 1.475, and 1.317 Å, respectively. Note that 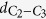 is between the bond lengths (1.420 and 1.544 Å) of graphite and diamond which have 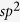 and 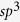 bonds, respectively. Because of a mixed bonding of 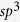 and 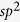, 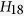 carbon has five different bond angles of 107.5°, 110.1°, 110.4°, 115.2° and 129.6°, contrasting with 109.5° for 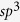 hybridized diamond and 120° for 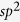 hybridized graphite. Such several bond distortions in 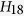 carbon implies the presence of strain, leading to a decrease of the relative stability compared to diamond and graphite as discussed below. In Table 1, we list the calculated lattice parameters, equilibrium densities, bond lengths, and cohesive energies of diamond, graphite, Rh6, T6, BC8, and H_18_ carbons. We find that our results agree well with previous DFT calculations and experiments^21^,^22^,^37^. We note that the equilibrium bulk atom density of H_18_ carbon is 3.135 g/cm^3^, larger than that (2.28 g/cm^3^) of graphite^21^.

In order to check the stability of H_18_ carbon, we perform the analyses of total energy, phonon mode, and elastic constants as well as molecular dynamic (MD) simulations.

**Figure 2:**
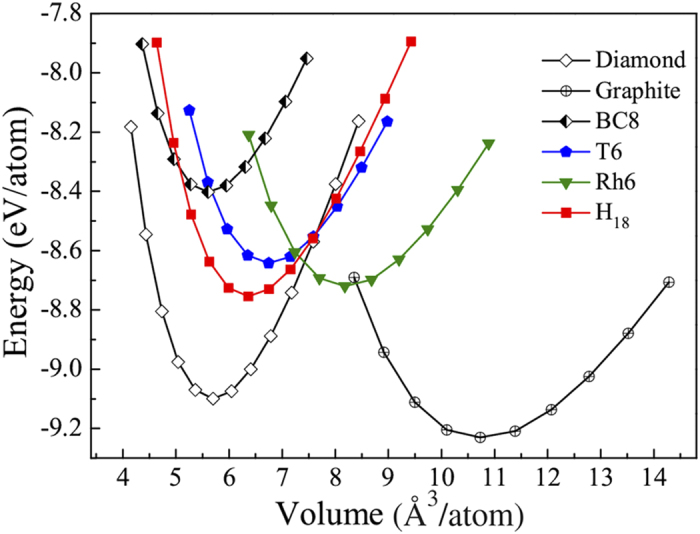
Total energy of H_18_ carbon as a function of volume per atom in comparison with those of diamond, graphite, BC8, T6, and Rh6 carbon (GGA-PBE calculations).

**Figure 3:**
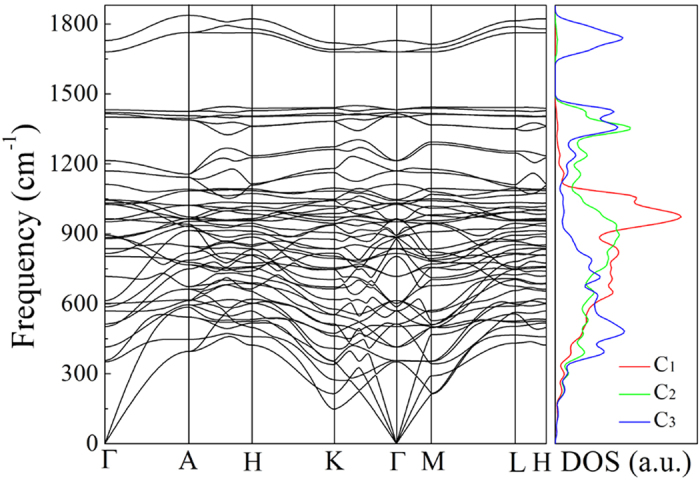
Calculated phonon band structures and density of states (DOS) of H_18_ carbon using the LDA calculations.

**Figure 4:**
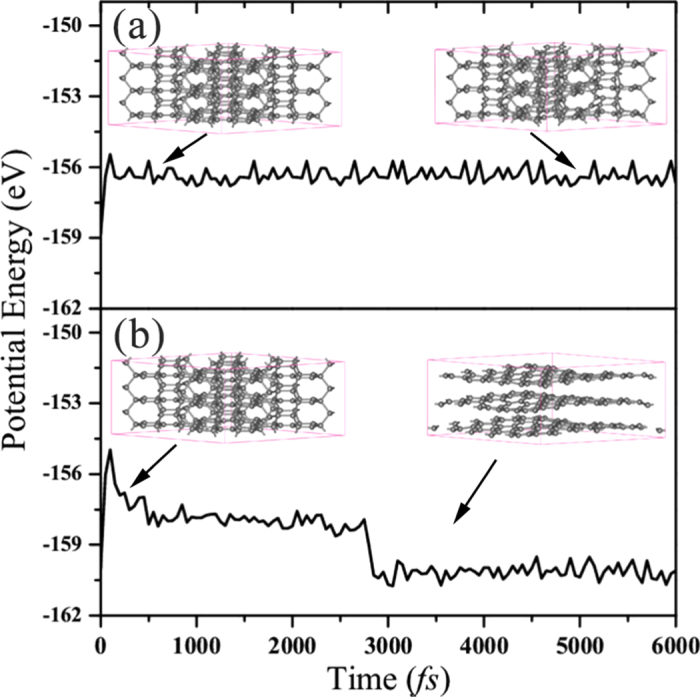
Potential energy fluctuation of H_18_ carbon in MD simulation at 1000 K (**a**) and 1500 K (**b**). (Inset) Snapshots depict the process of phase transition.

(i) *Total energy*: Figure 2 shows the calculated total energies of H_18_ carbon, diamond, graphite, BC8, T6 and Rh6 carbon as a function of volume per atom. It is seen that graphite and diamond are more thermodynamically stable than H_18_ carbon as well as other carbon phases. We note, however, that H_18_ carbon is not only much more stable than BC8 carbon (which has been suggested to be the derivative of cubic diamond under pressure of ∼1100 GPa^48^,^49^,^50^), but also more stable than T6 carbon and Rh6^20^,^37^. In particular, 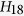 carbon has a relatively smaller 18-atom hexagonal unit cell compared to Rh6 (see Fig. 2). From the energy-volume curves of carbon allotropes (Fig. 2), one expects that Rh6 carbon can be transformed into H_18_ carbon by applying a certain pressure.

(ii) *Phonon mode*: The calculated phonon band structure and DOS are displayed in Fig. 3. It is found that there is no negative frequencies throughout the entire Brillioun zone, indicating the dynamical stability of 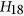 carbon. In Fig. 3, the highest vibrational frequency of H_18_ carbon amounts to ∼1837 cm^−1^ at *A* point, which is higher than ∼1400 cm^−1^ of the perfectly 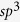 bonded diamond^51^ as well as ∼1600 cm^−1^ of the π-conjugated graphite^52^. We note that there exists a wide band gap of ∼230 cm^−1^ between 1449 and 1679 cm^−1^ near the *K* point. These features of phonon spectra of H_18_ carbon are anticipated to be measured by future experiments. In Fig. 3, we also plot the atom-resolved phonon DOS contributed by C_1_, C_2_ and C_3_ atoms, respectively. Obviously, the highest frequency modes around 1750 cm^−1^ originate from the vibrations of the strong 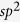 C_3 _− C_3_ bonds with a bond length of 1.317 Å, while the second highest frequency modes around 1450 cm^−1^ arise from the mixed 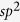-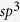 C_2_ − C_3_ bonds with a bond length of 1.475 Å. These characters of H_18_ carbon are different from both graphite with bond length of 1.420 Å and diamond with bond length of 1.544 Å.

(iii) *Elastic constants:* In the linear elastic regime, the elastic constant tensor is constituted a symmetric 6 × 6 matrix with 21 independent components, where only C_11_, C_12_, C_13_, C_33_ and C_44_ are independent in the hexagonal lattice^53^. According to the Born stability conditions^53^, the elastic constants of the hexagonal lattice should satisfy 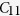 > 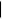

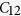

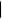
, 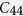 > 0, 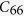 > 0, and 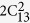 < C_33_ (C_11_ + C_12_). The calculated elastic constants of H_18_ carbon as well as diamond, T6, BC8, and Rh6 carbons are listed in Table 2. Specifically, the calculated elastic constants C_*ij*_ of H_18_ carbon satisfy well all of the conditions, indicating that the structure of H_18_ carbon is mechanically stable. The bulk modulus and shear modulus obtained by Voigt-Reuss-Hill approximation^54^ are also listed in Table 2. The magnitude of the bulk modulus of H_18_ carbon is fairly large as 360 GPa, which amounts to about four fifths of that (435 GPa) of diamond and even larger than that (337 GPa) of T6 carbon^37^. This result indicates that H_18_ carbon is more resistant to hydrostatic compression compared to T6 carbon. The B/G ratio of H_18_ carbon is about 1.0, close to that (0.83) of diamond, implying that H_18_ carbon can be characterized as a brittle material according to the Paugh criterion^55^.

(iv) *MD simulations*: To examine the thermal stability of H_18_ carbon, we carried out the ab initio MD simulations with the canonical (NVT) ensemble at temperature of 300, 1000 and 1500 K, respectively. The system is modeling with a 2 × 2 × 3 supercell containing 216 carbon atoms and the time step of 1 fs is used. The potential energy fluctuation of H_18_ carbon in MD simulation at 1000 and 1500 K are presented in Fig. (4a,b), respectively. We can see that the potential energy fluctuation are very small and geometry of H_18_ carbon remains intact after heating up to 1000 K for 6 picoseconds. With the temperature increasing up to 1500 K, we find that the H_18_ carbon becomes graphitization gradually (see in Fig. (4b)). These results have indicated that H_18_ carbon, once synthesized, can sustain the structure even at temperature of 1000 K. For comparison, Ab initio MD simulations for T6 carbon with the same setting and a 2 × 4 × 4 supercell containing 192 carbon atoms show that it is unstable even at 500 K^37^. Based on our MD simulations, we can say that H_18_ carbon is much more stable than T6 carbon at high temperature.

**Figure 5:**
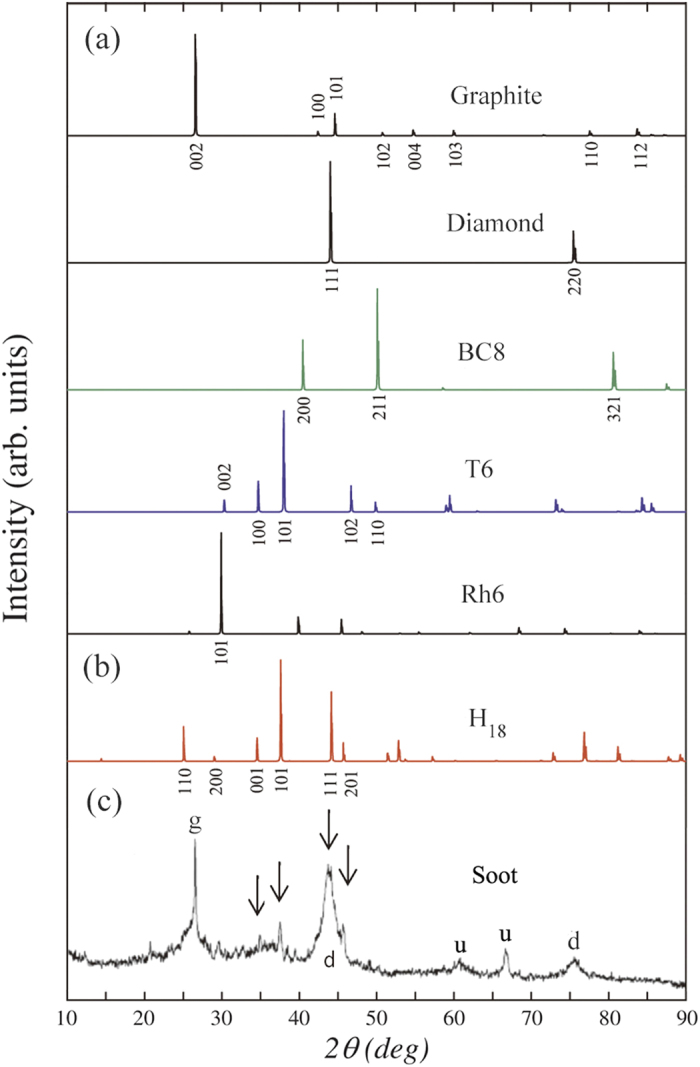
Comparison of simulated and experimental X-ray diffraction (XRD) patterns. **(a)** Simulated XRD patterns for graphite, diamond, BC8, T6 and Rh6 carbon. **(b)** Simulated XRD patterns for H_18_ carbon. **(c)** Experimental XRD patterns for detonation soot (sample Alaska C)^47^. 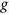, 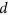, 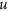 indicate graphite, diamond, unknown-carbon, respectively. The X-ray wavelength we adopted is 1.54059 Å.

In addition, to evidence the experimental observation of H_18_ carbon, we plot the simulated XRD spectra of graphite, diamond, BC8, T6, Rh6, and H_18_ carbons in Fig. 5(a,b), together with the experimental XRD data of TNT/RDX detonation soot in Fig. 5(c). In the experimental XRD data^47^, the diffraction lines arising from graphite (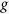), diamond (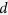), and other unknown (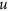) carbon phases were reported. As shown in Fig. 5(b,c,i) the (001), (101) and (201) peaks of H_18_ carbon match well with the experimental XRD spectra located at 34.4°, 37.4° and 45.5°, respectively, and (ii) the (111) peak of H_18_ carbon that overlaps with the (111) peak of diamond can be associated with the second-strongest experimental XRD peak at 43.9°. Meanwhile, the (110) and other peaks of 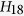 carbon can not be clearly identified in the experimental XRD patterns, but may be overlapped with neighboring peaks of other carbon phases. Note that the position of (100) and (101) peaks of T6 are very close to the position of (001) and (101) peaks of 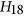 carbon, respectively. Since H_18_ carbon is thermodynamically more stable than T6 carbon as mentioned above, it is most likely that the experimental XRD peaks near 34° and 37° would be predominantly associated with H_18_ carbon rather than T6 carbon. Therefore, we propose that 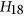 carbon is one of the unidentified carbon phases which were explicitly present in recent detonation experiments^47^.

**Figure 6:**
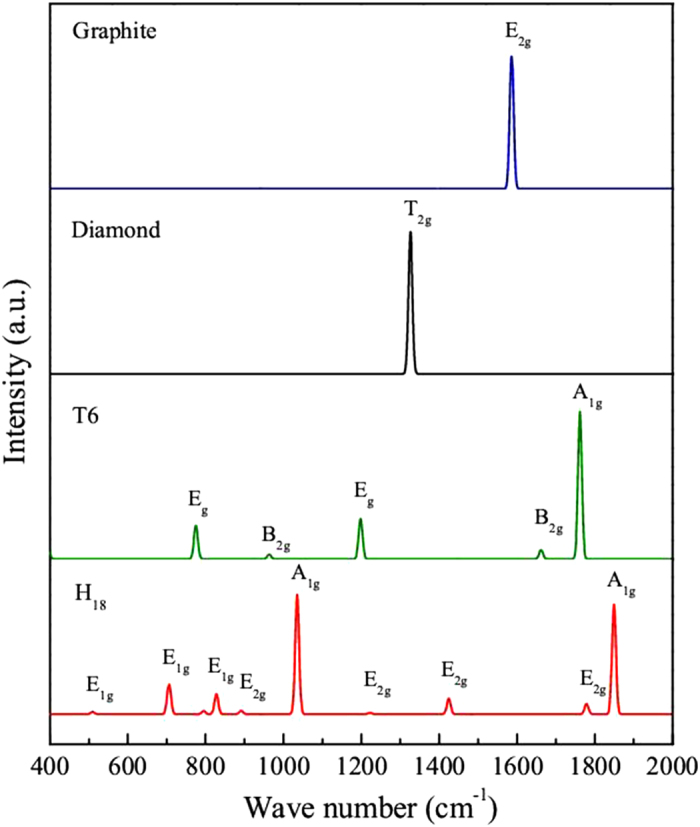
Comparison of simulated Raman spectra for *H*_18_, graphite, diamond and T6.

To provide more physical quantities that are accessible from experiments, we also simulated the Raman spectra of 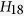 carbon and compared the results with graphite, diamond and T6 carbon structures. The simulation results are presented in Fig. 6. From Fig. 6, we can see that the E_2*g*_ mode in graphite is estimated to be 1586 cm^−1^, which is well agreement with the experimental data of 1581 cm^−1^ 56. The T_2*g*_ mode in diamond is estimated to be 1326 cm^−1^, which is close to the experimental data of 1333 cm^−1^ 57. Although the main XRD peaks of T6 and 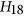 carbon are close to each other, their Raman spectra show rather different characters. For T6 carbon, there is only one main peak A_1*g*_ presents at 1762 cm^−1^. However, we find that, for 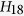, there are two main peaks A_1*g*_ at 1035 and 1849 cm^−1^, respectively. In addition, both T6 and 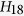 carbon also show some weaker peaks (E_*g*_ and B_2*g*_ for T6, E_1*g*_ and E_2*g*_ for 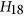). All the features above may be helpful for identifying this new carbon phase in further experiments.

**Figure 7:**
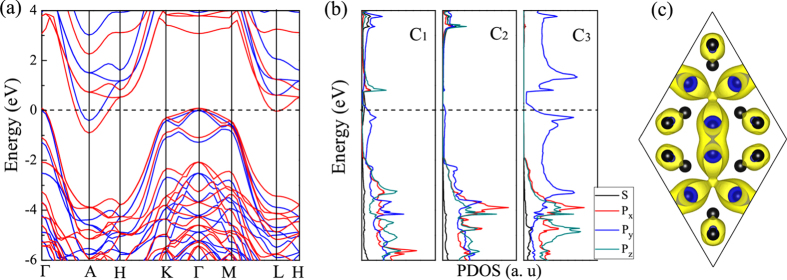
Electronic band structures, density of states and decomposed charge density of H_18_ carbon. **(a)** Electronic band structures calculated using GGA-PBE (red lines) and HSE06 hybrid functional (blue lines). **(b)** The projected density of states (PDOS) for C_1_, C_2_, and C_3_ atom at GGA-PBE level. The Fermi level is set at 0 eV. **(c)** The charge density isosurfaces (0.01 e/Å^3^) of the partially occupied band of H_18_ carbon.

Finally, we discuss the electronic properties of H_18_ carbon. The band structures and partial density of states (PDOS) are displayed in Fig. 7(a,b), respectively. It is seen that the Fermi level crosses the bands near the *A* and 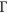 points, giving rise to the presence of the electron (hole) pocket around *A* (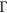). Thus, H_18_ carbon is metallic. It is noted that the metallic feature obtained using the semilocal DFT calculation with the Perdew-Burke-Ernzerhof (PBE) functional is preserved in the hybrid DFT calculation with the Heyd, Scuseria, and Ernzerhof (HSE06) functional^58^,^59^ [see Fig. 7(a)]. From the PDOS projected onto C_1_, C_2_ and C_3_ atoms [Fig. 7(b)], we find that the electronic states near 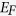 dominantly involve the 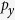 character of C_3_. In order to elucidate the origin of the metallic feature in H_18_ carbon, we calculate the charge density of the partially occupied bands in the energy windows of 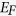 −0.5 to 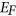 + 0.5 eV [see Fig. 7(c)]. It is seen that the 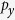 orbital of C_3_ atoms largely contribute to the charge density, forming a delocalized network. On the basis of the PDOS [Fig. 7(b)] and the charge character near 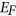 [Fig. 7(c)], we can say that the metallicity of H_18_ carbon is attributed to a large delocalization of the 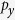 orbital of C_3_ atoms with 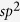 hybridization.

## Conclusion

Our first-principles DFT total energy and phonon calculations discover a novel stable carbon allotrope (termed H_18_ carbon) which is metallic. The analyses of the total energy, phonon mode, and elastic constants as well as molecular dynamic simulations obviously show that this new carbon allotrope exists as a stable structure. More importantly, we demonstrate that the H_18_ carbon may be one of the candidates of the unidentified carbon phases which appeared in the XRD spectrum analysis of a recent detonation experiment. In particular, 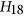 carbon has a metallic feature mainly due to the 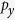 orbitals of 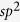 hybridized carbon atoms. Unlike previously reported 3D metallic carbon allotropes, 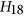 carbon can not only keep its metallicity at ambient pressure but also can likely maintain its structure at high temperatures. This novel 3D metallic carbon phase is anticipated to be useful for practical applications such as electronics and mechanics devices. Our findings will attract immediate broad interest and stimulate further experimental and theoretical studies for this new carbon allotrope.

## Methods

The present total-energy and phonon calculations were carried out by using the density functional theory. Both local density approximation (LDA) in the form of Ceperley-Alder^39^ and the generalized gradient approximation (GGA) developed by Perdew, Burke, and Ernzerhof (PBE)^40^ are adopted for the exchange-correlation potential as implemented in the Vienna 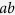 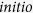 simulation package (VASP)^41^,^42^,^43^. All the discussions in this paper are based on the results got by GGA-PBE method, except for special notations. The all-electron projector augmented wave (PAW)^44^ method is adopted with C 2
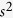
2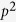 treated as valence electrons. A plane-wave basis set with an energy cutoff of 800 eV is used. The Gaussian smearing with a smearing factor of 0.05 eV is used in the calculations. The Brillouin zone (BZ) is sampled by a 9 × 9 × 21 Monkhorst-Pack (MP) special 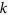-point grid including 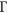-point. The geometries are optimized with no symmetry constraints, the convergence criteria employed for both the electronic self-consistent relaxation and the ionic relaxation are set to 10^−7^ eV and 10^−3^ eV/Å for electronic energy and Hellmann-Feynman force, respectively. Phonon dispersion curves are calculated by using the package phonopy^45^,^46^ with the forces calculated with VASP code. The first-principles molecular dynamics simulations are performed in the canonical (NVT) ensemble with the Nosé thermostat^60^. Each simulation lasted for 6 ps, with a time step of 1 fs. All the calculations in this work are performed at zero pressure.

## Additional Information

**How to cite this article**: Zhao, C.-X. *et al.* *H*_18_ Carbon: A New Metallic Phase with *sp*^2^-*sp*^3^ Hybridized Bonding Network. *Sci. Rep.* **6**, 21879; doi: 10.1038/srep21879 (2016).

## Figures and Tables

**Table 1:** Calculated lattice parameters *a* and *c* (in Å), equilibrium density (*ρ* in g/cm^3^), bond length (*d* in Å) and cohesive energy (E_*coh*_ in eV) of H_18_ carbon in comparison with those of diamond, graphite and some other carbon allotropes.

Structure	Space group	Method	a (Å)	c (Å)	*ρ* (g/cm^3^)	*d* (Å)	*E*_*coh*_ (eV/atom)
Diamond	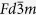	LDA	3.535		3.611	1.531	8.927
		PBE	3.572		3.501	1.578	7.780
		Exp.^22^	3.567		3.520	1.544	
Graphite	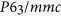	LDA	2.446	6.594	2.335	1.412	8.918
		PBE	2.467	8.145	1.859	1.424	7.900
		Exp.^21^	2.460	6.704	2.280	1.420	
T6	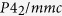	LDA	2.567	5.937	3.059	1.522, 1.331	8.416
		PBE^37^	2.600	6.000	2.952	1.540, 1.340	
BC8	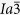	LDA	4.426		3.681	1.446, 1.611	8.239
		PBE	4.477		3.556	1.456, 1.631	7.082
Rh6	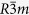	LDA	6.914	3.277	2.647	1.469, 1.357	8.441
		PBE	6.885	3.585	2.439	1.491, 1.357	7.400
H_18_	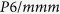	LDA	7.056	2.563	3.248	1.310–1.609	8.547
		PBE	7.125	2.605	3.135	1.317–1.631	7.269

**Table 2:** Calculated elastic constants C_*ij*_ (GPa), bulk modulus B (GPa), shear modulus G (GPa) and B/G value for H_18_ carbon in comparison with diamond, T6, BC8, and Rh6 carbon.

Structure	C_11_	C_12_	C_13_	C_33_	C_44_	C_66_	B	G	B/G
Diamond^23^	1055	126			565		435	522	0.83
T6^37^	730	25	89	1165	81	68	337	197	1.71
BC8	1172	54			559		427	559	0.76
Rh6	735	152	161	175	218		231	188	1.23
H_18_	1233	104	16	667	214		360	361	1.00
